# Recent advances in the mechanisms, current treatment status, and application of multifunctional biomaterials for radiation-induced skin injury

**DOI:** 10.7150/thno.108309

**Published:** 2025-01-27

**Authors:** Yang Xu, Quanying Liu, Wenfeng Li, Zhihe Hu, Chunmeng Shi

**Affiliations:** Institute of Rocket Force Medicine, State Key Laboratory of Trauma and Chemical Poisoning, Third Military Medical University (Army Medical University), Gaotanyan Road Street, Shapingba district, Chongqing 400038, China.; These authors contributed equally to this work: Yang Xu, Quanying Liu.

**Keywords:** Multifunctional, Biomaterials, Radiation-induced skin injury, Wound healing

## Abstract

Radiation-induced skin injury (RISI) is a prevalent complication following nuclear accidents and radiotherapy for tumors. The associated side effects may include erythema, desquamation, ulceration, and in severe cases, necrosis of certain skin tissues. These adverse reactions significantly impact the quality of life for patients and contribute to both psychological distress and economic burdens. However, there is currently no standardized protocol for the treatment and management of RISI. In comparison to traditional pharmaceuticals, the utilization of biomaterials in addressing radiation-induced diseases has garnered increasing attention due to their superior biocompatibility and outstanding functionality. Nevertheless, comprehensive reviews on this topic remain scarce. In this context, this paper systematically elucidates the pathogenesis of RISI, subsequently introducing the clinical manifestations and advancements in treatment for RISI. It emphasizes a comprehensive discussion on the design and innovation of novel biomaterials aimed at treating and protecting against RISI, while also illustrating the mechanisms by which multifunctional biomaterials enhance both treatment efficacy and protective measures for radiation-induced skin conditions. Finally, it addresses the challenges encountered by multifunctional biomaterials in managing radiation-related diseases and outlines potential directions for future research efforts. The objective of this review is to investigate the therapeutic and protective effects of multifunctional biomaterials in relation to radiation-induced skin injury, thereby providing significant reference value for the design and clinical application of innovative materials.

## 1. Introduction

The widespread utilization of nuclear technology across various sectors, including industry, medicine, science and technology, and the military, heightens the potential for nuclear accidents and radiation-related harm 1,2. The skin is the largest organ in the human body, serving multiple functions including the regulation of immunity and metabolism. It acts as the primary barrier against external threats, making it particularly vulnerable to damage 3. Radiation-induced skin injury (RISI) is a common complication associated with nuclear accidents and tumor radiotherapy 4. For instance, in the context of tumor radiotherapy, despite significant advancements in the precision and standardization of radiation therapy, the incidence of RISI is gradually on the rise. Approximately 95% of patients undergoing radiotherapy for tumors experience varying degrees of skin damage. Adverse reactions may present as erythema, peeling skin, and in severe cases, ulcers or even necrosis of skin tissue 5. These complications not only severely impact patients' quality of life but also impose substantial psychological stress and economic burdens. Furthermore, they can interfere with ongoing treatment regimens and disrupt the overall process of radiation therapy 6. Therefore, it is imperative to identify effective strategies for the treatment or prevention of skin damage resulting from ionizing radiation 7.

The normal process of skin wound healing typically progresses through four distinct stages: hemostasis, inflammation, proliferation, and remodeling 8. In contrast to standard wounds, radioactive skin lesions induce DNA damage and result in elevated levels of reactive oxygen species (ROS) production 9. With the accumulation of irradiation time and dose, the clinical and pathological manifestations of RISI may include, but are not limited to, dry and wet desquamation, erythema, edema, abnormal pigmentation. In severe cases, skin ulcers and fibrosis can occur, potentially leading to secondary infections that adversely affect the patient's overall health 10. Currently, there is no standardized gold standard treatment for RISI in clinical practice, and the existing research lacks universality. To date, several commercially available topical ointments have emerged, primarily composed of small molecular drugs that chemically neutralize toxic free radicals within skin cells. However, their effectiveness is significantly limited by unsatisfactory solubility, poor chemical stability, susceptibility to inactivation during long-term storage, restricted capacity to eliminate free radicals, and notable side effects. Consequently, the availability and practicality of these treatments are severely compromised 11. Addressing these limitations is crucial for enhancing the therapeutic efficacy of RISI. In light of these challenges, there exists an urgent necessity to develop a novel strategy aimed at improving both radiation protection and treatment effectiveness for the skin.

In recent years, multifunctional biomaterials have increasingly emerged as a significant focus of research in the field of regenerative medicine. Compared to traditional pharmaceuticals, these materials are extensively utilized for tissue repair and regeneration due to their advantages in biocompatibility, bioactivity, chemical stability, and degradability 12. Functional biomaterials not only exhibit structural and compositional similarities to the extracellular matrix, thereby promoting cell proliferation and migration while regulating the local microenvironment, but they also function as effective carriers for cells, drugs, or bioactive substances. This capability enables targeted delivery to specific sites, ultimately enhancing therapeutic outcomes 13. The researchers have discovered that these multifunctional biomaterials have significantly advanced in the protection and treatment of radiation-induced skin disorders. Therefore, it is crucial to integrate and interpret current research developments in this field to provide clinical guidance for the treatment and prevention of radiation-related illnesses.

Considering the rapid growth of interest in this field and the pressing need for effective treatments, this paper systematically elucidates the pathogenesis of RISI, subsequently introducing the clinical manifestations and advancements in treatment for RISI. It emphasizes a comprehensive discussion on the design and innovation of novel biomaterials aimed at treating and protecting against RISI, while also illustrating the mechanisms by which multifunctional biomaterials enhance both treatment efficacy and protective measures for radiation-induced skin conditions. Finally, it addresses the challenges encountered by multifunctional biomaterials in managing radiation-related diseases and outlines potential directions for future research efforts. This work aims to encourage and inspire a greater number of researchers to advance the design of innovative biomaterials for the treatment and prevention of radiation-related diseases. It seeks to foster the development of new radiation protection agents and aspires to facilitate the translation of relevant research into clinical applications, thereby realizing their potential value in practice.

## 2. Mechanisms of radiation-induced skin injury

**Figure 1:**
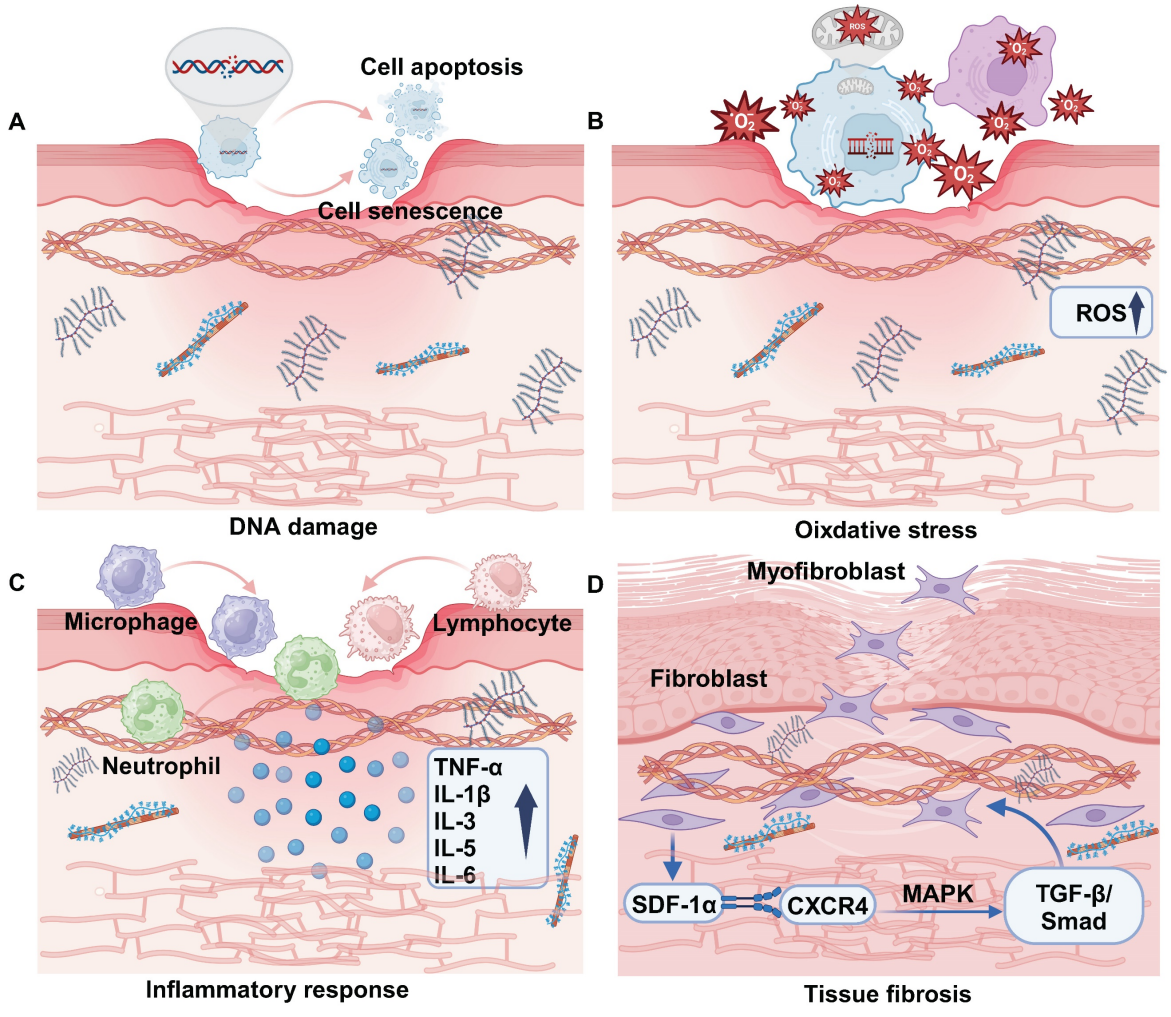
Pathogenesis of radiation-induced skin injury. (A) DNA damage. (B) Oxidative stress. (C) Inflammatory response. (D) Tissue fibrosis.

The mechanism of RISI is intricate. Currently, it is widely accepted that ionizing radiation can induce both reversible and irreversible damage to the DNA of biological tissues and cells. This damage may lead to DNA mutations and structural abnormalities, affecting gene transcription and protein levels, potentially resulting in cell death. Additionally, ionizing radiation triggers the production of highly reactive molecules such as hydroxyl free radicals, which attack proteins, lipids, and DNA. This process activates various cellular signaling pathways that result in the release of inflammatory factors, contributing to cellular and tissue damage **(Figure 1)** 14. Simultaneously, blood vessel impairment caused by ionizing radiation disrupts the normal supply of nutrients, exacerbates tissue hypoxia, and promotes fibrosis process 15. As a result, the prolonged inflammatory response coupled with ongoing cellular damage and repair processes leads to excessive activation of fibroblasts. This results in abnormal deposition of collagen and extracellular matrix proteins, rendering the skin difficult to heal and causing it to appear erythematic, swollen, and ulcerated.

### 2.1 DNA damage

DNA is an organic molecule that serves as a crucial repository of genetic information essential for the development and maintenance of life. Its chemical properties and structure can be readily altered under the influence of ionizing radiation (IR). DNA damage encompasses both physical and chemical alterations that directly or indirectly compromise DNA strands, including single-strand breaks, double-strand breaks, base deletions, and cross-linking damage **(Figure 1A)** 16. These lesions can be identified using a range of techniques, including single-cell gel electrophoresis and pulsed-field gel electrophoresis. DNA damage occurs *in vivo* and is repaired through various mechanisms such as base excision repair, nucleotide excision repair, mismatch repair, homologous recombination repair, and non-homologous end joining. These processes work together to mitigate the potentially harmful effects of DNA damage 17. When repair mechanisms are compromised, a significant accumulation of DNA damage occurs within the organism, resulting in cellular senescence, apoptosis, or mutations that adversely affect skin integrity 18.

Notably, radiation-induced senescent cells, despite their inability to undergo cell division and being permanently arrested in proliferation, consistently demonstrate apoptotic resistance, metabolic activity, and the secretion of pro-inflammatory and pro-fibrotic molecules. Additionally, they induce changes in the adjacent microenvironment. Among these effects, the bystander effects associated with ionizing radiation-induced cellular senescence are primarily mediated by exosomes and microRNAs (cells that are not directly exposed to ionizing radiation also exhibit senility-like features when they receive signaling molecules released by irradiated cells) 19. For example, exosomes derived from senescent cells are enriched with a variety of inflammatory mediators, such as IL-6, IL-8, TNF-α, etc. These factors have the potential to activate inflammatory signaling pathways in surrounding healthy cells, inducing them to enter a chronic low-grade inflammatory environment similar to the state of aging. Additionally, they may disrupt normal cellular metabolism by impairing mitochondrial function or elevating levels of ROS 20. At the same time, a significant number of microRNAs (miRNAs) implicated in the aging process have been identified, and their alterations can lead to DNA damage, γH2AX lesion formation, increased ROS, and oxidative stress. For instance, miR-34a promoted cellular senescence by targeting SIRT1 and downregulating its expression, which subsequently activated p53 21. The overexpression of miR-21 has been shown to diminish the replicative lifespan of human umbilical vein endothelial cells (HUVECs) 22. Additionally, miR-22 facilitated cellular senescence in human fibroblasts and epithelial cells by targeting CDK6, Sp1, and SIRT1 23. Currently, the p16-pRB and p53-p21 pathways are widely recognized as significant mechanisms underlying radiation-induced aging 24. It has been reported that mitochondrial dysfunction, ferritin autophagy, and the inflammation cascade induced by cyclic guanosine monophosphate (cGMP)-adenosine monophosphate (AMP) synthase (cGAS) may also serve as potential mechanisms contributing to radiation-induced aging 25. Our research team has identified human positive cofactor 4 (PC4) as a potential target in the context of cellular aging. PC4 accumulated and became activated during the aging process, contributing to its acceleration by disrupting mTOR-regulated protein homeostasis 26. The overall process by which ionizing radiation induces DNA damage remains complex and is not yet fully elucidated. Nevertheless, research indicated that emerging techniques in DNA nanotechnology and atomic force microscopy (AFM) hold promise for uncovering the fundamental mechanisms underlying DNA damage 9.

### 2.2 Oxidative stress

Ionizing radiation can inflict damage on DNA either directly or indirectly, primarily through the radiolysis of water, which generates free radicals that subsequently attack cellular membrane lipids, proteins, and DNA. This process leads to oxidative stress and ultimately results in cellular damage. Mitochondria are organelles tasked with energy production within cells and play a crucial role in the regulation of oxidative stress 27. Free radicals generated by ionizing radiation enhance the expression of cyclooxygenases (COXs), nitric oxide synthases (NOS), lipoxygenases (LOX), and NADPH oxidase, thereby impacting mitochondrial function 28. Increased levels of ROS lead to mitochondrial dysfunction, which is typically characterized by impaired respiratory chain activity, structural abnormalities, depletion of the cellular ATP pool, disruption of cell signaling pathways, and an elevated production of mitochondria-derived ROS (mtROS)** (Figure 1B)** 29. These mtROS may undermine the integrity of mitochondrial membranes, leading to the release of mitochondrial ligands or damage-associated molecular patterns (DAMPs), which can further exacerbate mitochondrial dysfunction 28. At the tissue level, the accumulation of ROS and oxidative stress can disrupt endothelial cell connections, resulting in damage to vascular endothelial cells. This process increases permeability and further compromises vascular epithelial function, ultimately leading to tissue hypoxia and necrosis 30. In radiation-induced skin injury, TGF-β binds to its receptor to up-regulate the expression of miRNA-21, thereby inhibiting the production of superoxide dismutase 2 (SOD2), which is an important factor in the elimination of ROS 31.

### 2.3 Inflammatory response

Ionizing radiation causes damaged cells to release inflammatory mediators that attract immune cells (including lymphocytes, macrophages, and neutrophils) to the damaged skin area. These cells release various cytokines and chemokines that further amplify the inflammatory response 32. The early inflammatory response is primarily characterized by an increase in pro-inflammatory cytokines (such as IL-1β, IL-3, IL-5, IL-6, and TNF-α) and chemokines. These mediators contribute to skin tissue injury and compromise barrier integrity by activating the local inflammatory responses of eosinophils and neutrophils **(Figure 1C)** 33. In addition, skin exposure to ionizing radiation induces cellular senescence of keratinocytes. These aging keratinocytes will not only over proliferate, leading to the destruction of the structural function of the epidermis, but also further secrete inflammatory mediators and cause radiation dermatitis 34. Cellular iron death induced by lipid peroxidation and the formation of ferritin in keratinocytes is also a contributing factor to necrotizing skin inflammation 35,36.

Further intensification of the inflammatory response is implicated in the activation and intranuclear migration of nuclear factor-κB (NF-κB). Initially, ionizing radiation induces an increase in intracellular ROS, which subsequently activates the IKK (IκB kinase) complex. This activation leads to the phosphorylation and degradation of IκB (inhibitory factor κB). Following the degradation of IκB, NF-κB is liberated from the cytoplasm and transferred into the nucleus. Within the nucleus, NF-κB binds to the promoter region of target genes, thereby promoting the expression of various inflammatory mediators (such as IL-1β, IL-6, TNF-α, etc.) and chemokines. The activation of ROS further extends to the NLRP3 pathway, facilitating the release of Caspase-1 and the extracellular secretion of pro-inflammatory cytokines such as IL-1β and IL-8. This cascade triggers inflammation and promotes apoptosis 37. Based on this, NLRP3 and NF-κB may be regarded as promising targets for mitigating radiation-induced skin inflammatory damage. This can be achieved by directly or indirectly inhibiting their upstream signaling pathways, thereby alleviating skin damage through the reduction of the inflammatory response. Due to the intricate nature of the skin immune response, further investigation into the interactions occurring among epithelial cells, stromal cells, and various immune cell populations is essential. This research will enhance our understanding of the mechanisms that regulate skin immune homeostasis and inflammation in the context of ionizing radiation 24.

### 2.4 Tissue fibrosis

Radiation-induced fibrosis is a pathological condition characterized by the persistent over-activation of fibroblasts, leading to the destruction of normal tissue components. This process results in an excessive accumulation of extracellular matrix, which can ultimately cause irreversible damage to the affected tissues 38,39. Skin fibrosis is a complex process that involves the intricate interactions among various cells and cytokines 40. Ionizing radiation facilitates the activation of myofibroblasts by engaging various signaling pathways, including TGF-β and Wnt/β-catenin. Additionally, it can stimulate fibroblasts, endothelial cells, and vascular smooth muscle cells, prompting their transformation into myofibroblasts 41. For instance, the interaction between stromal cell-derived factor-1α (SDF-1α) and its receptor CXCR4 activates the TGF-β/Smad signaling pathway through downstream MAPK signaling. This cascade ultimately contributes to the promotion of fibrosis **(Figure 1D)** 42. When activated myofibroblasts secrete alpha-smooth muscle actin (α-SMA) and types I, III, and IV collagen, integrins link the alpha-SMA stress fibers to collagen, creating high contractility and hardening the skin tissue microenvironment, which can eventually lead to its sclerosis and dysfunction 43. In addition, microvascular injury may lead to skin fibrosis. The exposure of subcutaneous extracellular matrix (ECM) components to platelets promotes the excessive secretion of von Willebrand factor (vWF) and platelet-activating factor (PAF), while simultaneously diminishing the production of nitric oxide (NO), prostacyclin, and the transmembrane glycoprotein thrombomodulin (TM), along with their fibrinolytic activity 44. These alterations ultimately trigger an anti-fibrinolytic response and initiate a clotting cascade, resulting in coagulation and vascular occlusion, which accelerates the process of fibrosis 45.

## 3. Clinical manifestation and treatment status of RISI

Radiation skin injuries can be categorized into acute and chronic types. Furthermore, the severity of these injuries can be classified to facilitate the development and implementation of appropriate treatment plans and measures 46. Acute radiation-induced skin injury refers to acute radiation dermatitis and radiation skin ulcer caused by multiple large doses of external radiation in one or a short period of time (a few days) 47. The pathophysiological changes of acute RISI mainly involve erythema, dry and wet desquamation, skin necrosis, ulceration, and bleeding, which usually occur within 90 days 48. According to the National Cancer Institute Common Terminology Criteria for Adverse Events (NCI CTCAE) version 3.0, acute radiation-induced skin injury can be classified into 5 grades from grade 0 to grade IV according to the degree of injury. The specific skin injury and manifestations are shown in **Table 1** 49. Different from acute RISI, chronic RISI is a delayed adverse reaction with long latency, obvious potential, progression, persistence and recurrence. Pathophysiological changes in chronic RISI involve symptoms such as pigmentation, chronic skin atrophy, ulcers, telangiectasia, fibrosis, and skin cancer 50. The grading standards for chronic radiation dermatitis are primarily based on the criteria established by the American Cancer Radiation Therapy Association Group. These standards typically categorize the condition into 6 grades ranging from 0 to V degrees **(Table 2)** 51.

RISI can significantly impact patients' quality of life and the overall effectiveness of treatment following radiation therapy. Consequently, the management of RISI has been a focal point for both clinicians and researchers. While numerous clinical approaches exist for the prevention and treatment of RISI, both domestically and internationally, many of these methods lack targeted and effective protocols.

### 3.1 Topical care

The management of RISI is an integral part of the entire radiation therapy process; however, there is currently no standardized approach to care. Traditional skin care encompasses the cleaning and maintenance of the patient's skin, which can effectively prevent wound infections, alleviate physical discomfort, and facilitate subsequent treatment for patients. The irradiated area of the patient's skin should be maintained in a clean and dry condition. Initially, it is essential to use a mild non-irritating cleanser to gently cleanse the affected skin; water temperature should not be excessively high, and alkaline soaps should be avoided 52. Following cleansing, a moisturizing cream containing vitamin E, hyaluronic acid, and other beneficial ingredients should be applied to keep the skin hydrated. This helps reduce external irritation while ensuring that wounds remain in a dry and well-ventilated state. It is also important to avoid using irritating dressings that could exacerbate the condition or lead to secondary damage from friction with clothing. It is crucial to recognize that excessive or improper washing can disrupt the biophysical properties of the skin barrier and may exacerbate symptoms of inflammation or injury. Therefore, it is essential to pay attention to both the duration and frequency of cleansing. It is recommended that patients clean their wounds once or twice daily, avoiding prolonged immersion during radiation therapy.

### 3.2 Drug therapy

The medications employed in the treatment of radiation injuries primarily consist of hormone therapies for anti-inflammatory purposes, vascular agents for antioxidant therapy, and superoxide dismutase 53. The inflammatory response resulting from acute RISI is typically managed with local corticosteroids. These agents exhibit therapeutic effects owing to their anti-inflammatory, immunosuppressive, anti-proliferative, and vasoconstrictive properties. For instance, they effectively inhibit cytokine surges induced by radiation, diminish the occurrence of wet desquamation, and postpone the onset of dermatitis 54. Statins possess anti-inflammatory, immunomodulatory, antioxidant, metabolic, and antimicrobial properties that can enhance the management of skin diseases and facilitate the healing of ulcerative wounds. Notably, they have been demonstrated to significantly alleviate radiation-induced swelling, itching, and pain 55. Melatonin is a potent free radical scavenger with notable antioxidant and anti-inflammatory properties, capable of maintaining mitochondrial homeostasis under various experimental conditions and exhibits significant anti-aging effects 56. Amifostine, recognized for its antioxidant capabilities, was the first clinical radioprotective agent approved by the United States Food and Drug Administration (FDA). However, its clinical application has been limited due to pronounced adverse reactions such as nausea, vomiting, and fatigue, as well as restricted routes of administration 57. Topical emulsions of triethanolamine have demonstrated efficacy in the treatment of radiation dermatitis. These formulations facilitate the removal of necrotic tissue, promote fibroblast proliferation, mitigate *in vitro* vascular alterations, restore CD34 expression, enhance epithelial cell proliferation, and decrease IL-1 secretion. Sucralfate is a widely used anti-ulcer medication when administered orally. In topical formulations, sucralfate has demonstrated significant barrier protection, antibacterial activity, anti-inflammatory effects, and the ability to promote angiogenesis. However, clinical outcomes regarding the efficacy of sucralfate in radioactive skin therapy have yielded mixed results. The largest and most rigorously conducted studies reported no significant impact of sucralfate on alleviating dermatitis severity or relieving symptoms in patients. Similar findings were presented by Wells *et al.* Conversely, Maiche *et al.* assessed that a 7% sugar sulfate cream could substantially mitigate acute radiation reactions affecting the skin 58. In addition, our research group has discovered that, compared to rapamycin, cordycepin effectively can inhibit cellular senescence through the activation of NRF2 and AMPK pathways. This finding suggested that cordycepin may offer a more effective treatment for alleviating radiation-induced skin ulcers and held promise as a potential palliative option for managing such conditions 59,60. Similarly, dasatinib plus quercetin was shown to alleviate radioactive skin ulcers by removing senescent cells 61. It is essential to recognize that the pharmacological management of RISI constitutes a common and significant intervention, capable of alleviating symptoms, promoting healing, and mitigating complications to varying extents **(Table 3)**. However, it is important to acknowledge that drug treatments may also lead to substantial side effects, and individual patients can exhibit considerable variability in their responses to the same medication. Therefore, we must consider diverse prevention and treatment strategies based on the underlying pathogenesis and select the most appropriate medication tailored to each specific case in order to achieve optimal outcomes.

### 3.3 Cytokines

Epidermal growth factor (EGF) plays a crucial role in promoting the proliferation of human epidermal stem cells, fibroblasts, and keratinocytes 62. Experimental studies have demonstrated that EGF, which is released by platelets, macrophages, and fibroblasts, plays a significant role in promoting wound healing through the process of skin re-epithelialization 63. There are clinical studies using recombinant human epidermal growth factor (rh-EGF) to treat radiation-induced chronic wounds for 16 weeks. In comparison to flap surgery and conventional treatments, rh-EGF has demonstrated significant efficacy in alleviating symptoms and promoting the healing process of chronic ulcer wounds 64. Fibroblast growth factor (FGF) can promote capillary regeneration and improve local blood circulation, and the healing time of RISI patients treated with FGF was shorter than that of conventional therapy 65. Recombinant human granulocyte-macrophage colony-stimulating factor (rhGM-CSF) can be effectively utilized in the management of refractory wounds following chemoradiotherapy. It demonstrates both local anti-infective properties and promotes wound healing 66. Chen *et al.* have discovered that senescent fibroblasts accumulate at the site of RISI and can modulate the local immune microenvironment through the secretion of IL-33. This process accelerated epithelialization and collagen deposition, thereby contributing to wound healing. These findings offered valuable insights for developing intervention strategies targeting senescent cells in RISI **(Table 3)** 67. Although the topical application of growth factors offers a relatively gentle and operationally simple therapeutic approach, the use of a single growth factor may have limited efficacy and is prone to inactivation *in vitro*, which could necessitate the combination of multiple growth factors and precise control of dose and timing of administration. However, there is still a lack of sufficient large-scale randomized controlled clinical trial evidence to comprehensively evaluate the effectiveness and reliability of this approach.

### 3.4 Surgical treatment

RISI can result in severe complications such as skin ulcers, necrosis, and infections. In cases where patients do not respond to conservative treatment or present with advanced conditions, surgical intervention becomes a crucial option. Some researchers have employed Doppler ultrasound to design axial skin flaps for the repair of radioactive ulcers on the chest wall in patients following radical breast cancer surgery.

Their findings indicated that these patients experienced successful flap survival, adequate blood circulation, and no recurrence of ulcers during postoperative follow-up. Pulsed dye laser therapy is primarily used to address abnormally dilated capillaries, thereby reducing erythema and inflammatory responses 68. Hyperbaric oxygen therapy primarily alleviates pain and improves wound healing by increasing local oxygen tension and enhancing angiogenesis 69. While surgical treatment can remove diseased tissue to prevent further deterioration of the condition, it is time-consuming, carries higher surgical risks, has a long recovery period, and requires a higher level of professional expertise from the surgeon.

The studies mentioned above all exhibit limitations in the treatment of RISI, particularly in their failure to classify patients based on their skin's sensitivity to radiation. Therefore, it is essential to determine whether the patient suffers from any conditions that may increase radiation sensitivity, such as diabetes and vascular diseases. Additionally, an initial assessment of the patient's radiation sensitivity can be made through direct observation of skin condition and minimal erythema dose testing, with findings documented 70. Further evaluation can be conducted by measuring specific biomarkers, such as DNA damage repair capacity and levels of cellular apoptosis, to quantify individual radiation sensitivity 71. Although we have identified genes that are altered in skin tissue following radiation exposure through high-throughput sequencing, defining truly "radiation-specific" genes requires additional research to confirm which genes are uniquely or predominantly activated or inhibited under radiation conditions while remaining relatively quiescent in other environments 72. This would provide a theoretical foundation and technical support for developing new strategies for radiation protection. Furthermore, current clinical approaches for managing RISI predominantly rely on pharmacological interventions. While the aforementioned drugs possess varying degrees of antioxidant and anti-inflammatory properties, they suffer from poor chemical stability and limited free radical scavenging capabilities. Consequently, satisfactory therapeutic outcomes have yet to be achieved. Most widely used traditional medicines primarily focus on alleviating symptoms rather than addressing underlying issues. Consequently, it is imperative to conduct comprehensive investigations aimed at identifying more effective prevention and control strategies.

## 4. Research progress of biomaterials in radiation-induced skin injury

**Figure 2:**
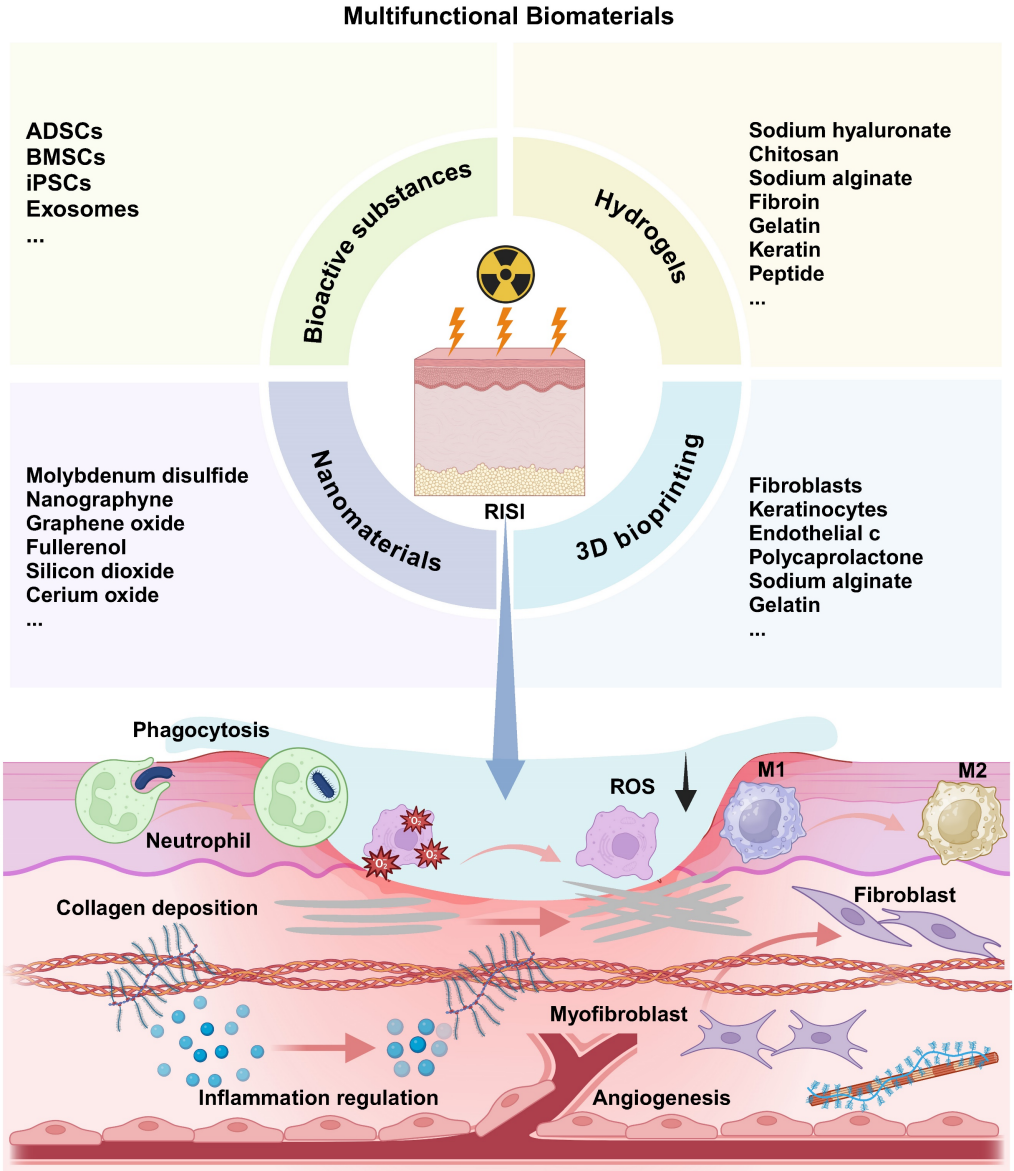
Multifunctional biomaterials for radiation-induced skin injury and its therapeutic mechanism.

In recent years, the utilization of biomaterials in the treatment of radiation-related diseases and in radiation protection has garnered increasing attention. Unlike traditional pharmaceutical compounds, biomaterials offer a range of advantages, including exceptional biological activity, chemical stability across various physiological conditions, superior biocompatibility, and targeted delivery capabilities **(Table 4)** 73. It is crucial to emphasize that the strategic integration of specific biomaterials with particular therapeutic agents can yield synergistic therapeutic effects, thereby paving new avenues for the treatment of radiation-induced ailments **(Figure 2)**.

### 4.1 Stem cells and exosomes

**Figure 3:**
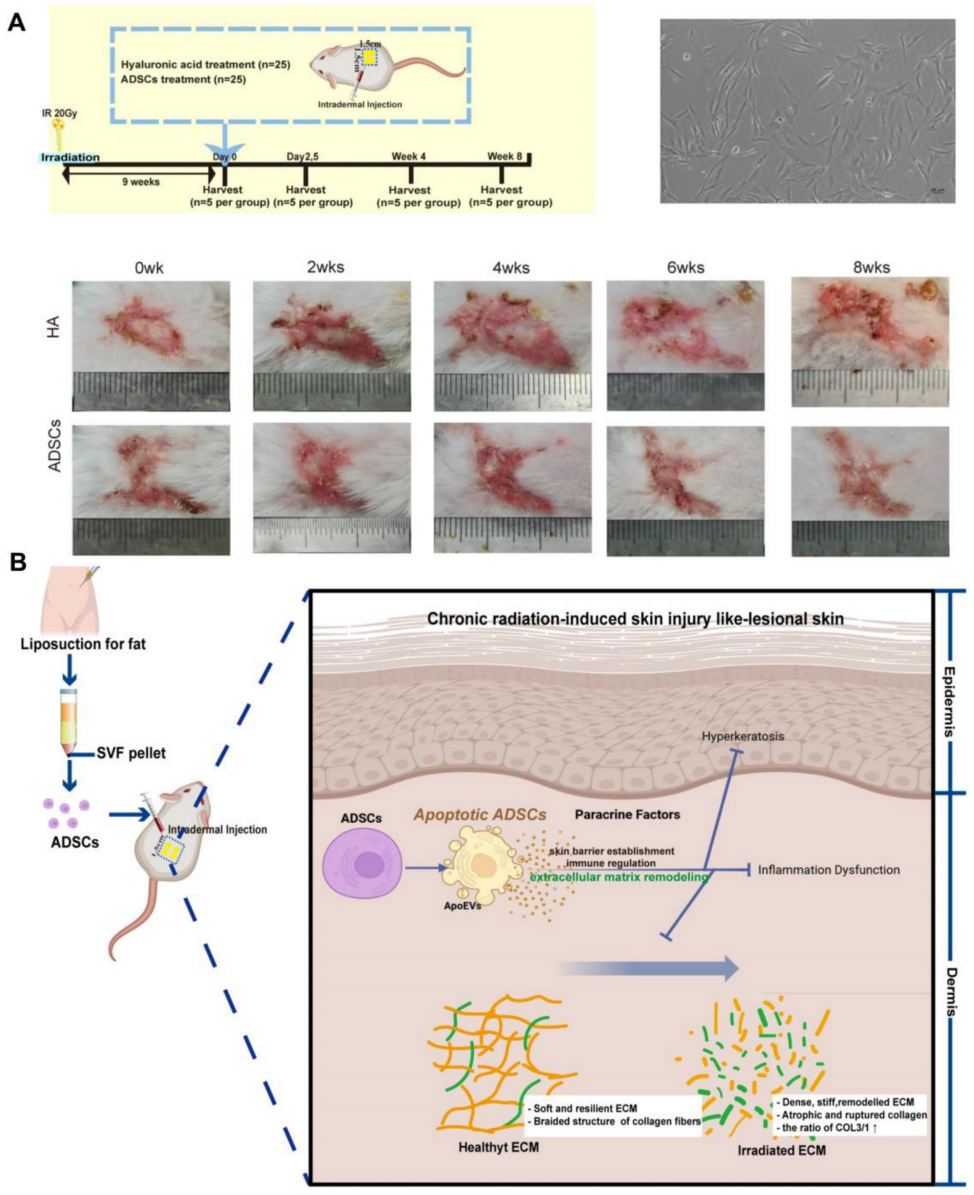
(A) Therapeutic effects of ADSCs on chronic radiation dermatitis skin regeneration. (B) Illustration of the therapeutic mechanisms of apoptotic ADSCs on chronic radiation dermatitis. Adapted with permission from 74, copyright 2023 Springer Nature.

Therapies utilizing stem cells have been documented to enhance wound healing following exposure to radiation 101. Zhu *et al.* discovered that adipose-derived stem cells (ADSCs) injected into the skin of patients suffering from chronic radiation dermatitis can significantly alleviate symptoms associated with this condition, including fibrosis and irregular collagen deposition. Furthermore, these cells promote the regeneration of skin affected by severe radiation damage. The proposed therapeutic mechanism may involve ADSCs undergoing apoptosis, which in turn facilitates the remodeling and reorganization of the dermal collagen matrix, thereby enhancing the vitality of radiation-damaged skin in murine models **(Figure 3)** 74. The researchers discovered that bone marrow mesenchymal stem cells (BMSCs) were also effective in alleviating skin inflammation and fibrosis in mice suffering from acute RISI 75. Human-induced pluripotent stem cells (iPSCs) demonstrate a positive impact on the healing of irradiated skin wounds by facilitating re-epithelialization and enhancing angiogenesis when compared to the PBS group 76. Although mesenchymal stem cells are known to effectively promote tissue repair following injury, there is a growing body of evidence suggesting that their therapeutic effects are primarily mediated through paracrine mechanisms. Consequently, exosomes derived from stem cells have recently emerged as a promising cell-free strategy for enhancing skin regeneration, thereby addressing the challenges associated with the immunogenicity of stem cells 102. Studies have demonstrated that microRNAs (miRNAs) derived from mesenchymal stem cell (MSC)-derived exosomes play a significant role in mitigating both acute and chronic radiation damage. For instance, miRNA-210 had been shown to facilitate DNA repair by modulating hypoxia-inducible factor 1 (HIF-1), promote the proliferation and migration of epithelial cells, and participate in immune responses. These findings suggested that irradiated cells may leverage MSC-derived exosomes miRNAs to enhance their resistance to ionizing radiation 103. And miRNA-21 inhibited apoptosis in vascular endothelial cells and mitigated ionizing radiation-induced vascular damage *in vitro* by targeting the PTEN gene which regulating the PI3K/AKT signaling pathway 104. Engineered exosomes have garnered significant attention due to their distinctive biological properties and versatility 77. These modified exosomes can be enhanced through the incorporation of drugs, growth factors, gene editing techniques, and various other modification methods to facilitate targeted therapeutic interventions 105,106.

Overall, both stem cells and exosomes play a pivotal role in tissue repair; however, their retention and stability *in vivo* present significant challenges 107. In light of these obstacles, hydrogels have emerged as reliable carriers for the delivery of bioactive substances. As a highly hydrophilic gel characterized by a unique three-dimensional network structure, hydrogels not only exhibit biocompatibility but also possess the ability to rapidly absorb and retain substantial amounts of water without dissolving. Furthermore, they are anticipated to offer specialized functions such as promoting blood coagulation, enhancing tissue adhesion, exhibiting antibacterial activity, providing anti-inflammatory properties, and demonstrating antioxidant effects. These functionalities are essential for facilitating the collaboration between stem cells and exosomes in promoting skin tissue regeneration and repair.

### 4.2 Intelligent responsive hydrogels

**Figure 4:**
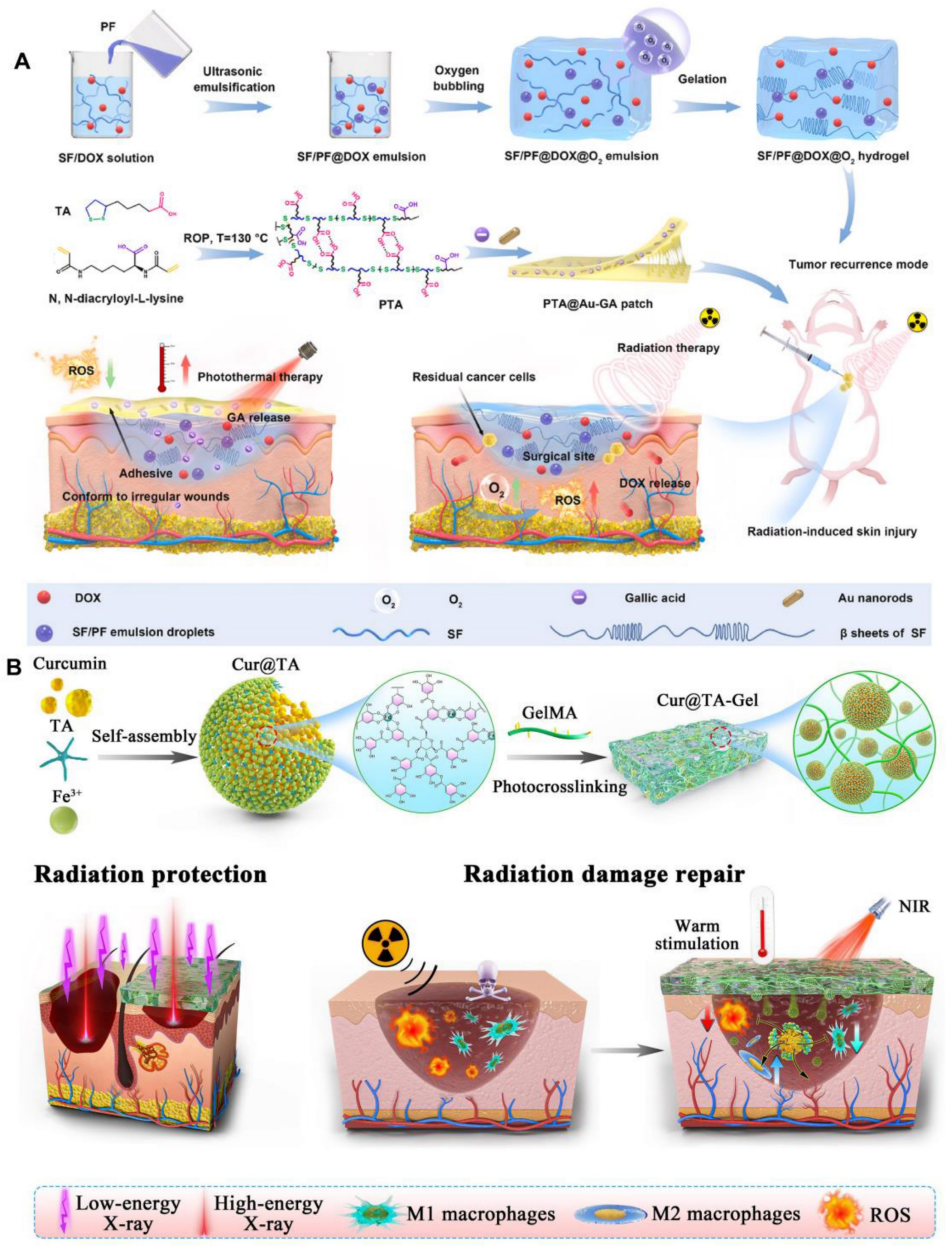
(A) Schematic illustration of the fabrication of SF/PF@DOX hydrogel and PTA@Au-GA patch and their application in treatment of breast cancer recurrence and radiation skin injury. Adapted with permission from 114, copyright 2024 Elsevier. (B) Schematic diagram illustrating of the preparation process of the nanohybrid hydrogel patch and the principle of its application in radiation protection and radiation dermatitis. Adapted with permission from 80, copyright 2023 Elsevier.

The environmental responsiveness of hydrogels represents an advanced drug delivery strategy that adapts to variations in external conditions, including temperature 108, photothermal effects 78, pH 109, hydrogen peroxide (H_2_O_2_) 110, enzymes 111, and glucose 112 etc. This capability enables the precise release of drugs at optimal times and targeted locations **(Table 5)** 113. Zhang *et al.* developed a biodegradable fibroin/perfluorocarbon (SF/PF) hydrogel loaded with doxorubicin (DOX), as well as an antioxidant bioadhesive containing gold nanorods (AuNRs) and gallic acid (GA). These innovative formulations are designed to effectively inhibit tumor recurrence and mitigate skin radiation damage by providing mild photothermal therapy alongside antioxidant properties **(Figure 4A)** 78,114. Wang *et al.* grafted the photosensitive group methacrylate anhydride (MA) onto chitosan (CHI) and gelatin (GEL), subsequently incorporating epigallocatechin gallate (EGCG) through physical means. The resulting composite photosensitive hydrogel exhibited excellent swelling properties, favorable rheological characteristics, and biocompatibility. Furthermore, it had been shown to promote angiogenesis and demonstrated a significant therapeutic effect on radiation skin injuries 79. An immunomodulatory sticky hydrogel patch, encapsulated with curcumin and tannic acid, exhibited tissue adhesion and photothermal properties. This innovative patch demonstrated a robust ability to clear ROS and facilitated the polarization of macrophages towards the M2 phenotype. It was effectively utilized for the repair of radiation dermatitis and provided radiological protection **(Figure 4B)** 80. By hybridizing the luminescent 3D terbium (III) metal-organic framework (Tb-MOF) with sodium alginate (SA) gel, utilizing terbium (III) as a crosslinking agent, researches successfully prepared Tb-MOF/Tb-AG hydrogels. These hydrogels exhibited multiple luminescence centers and an ample number of uranium binding sites, enabling rapid response to uranium detection. This innovative approach held promise for applications in human health 115. It had been reported that a novel composite hydrogel, developed from physically cross-linked hyaluronic acid, can effectively enhance the healing of skin ulcers induced by radiation. This was achieved through the application of freeze-thaw technology, which incorporated small molecule drugs such as deferoxamine and retinoic acid 32.

In addition, functional hydrogels demonstrate significant potential in the treatment of RISI. Keratin-based hydrogels can enhance wound healing by promoting cell proliferation and collagen synthesis following radiation exposure 85. Multifunctional peptide hydrogels that mimic the extracellular matrix effectively repair RISI by eliminating harmful ROS and facilitating angiogenesis 86,87. Glycopeptide hydrogels derived from glucomannan can mediate macrophage polarization towards the M2 phenotype, thereby improving the persistent inflammatory microenvironment associated with RISI. This approach significantly reduced epithelial hyperplasia and accelerated wound healing 88. The treatment of radiation-induced skin wounds in SD rats using sildenafil citrate hydrogel not only facilitated skin re-epithelialization and collagen deposition but also mitigated inflammatory infiltration 116. The ferulic acid hydrogel developed by Huang *et al.* has demonstrated a significant ability to reduce inflammation and oxidative stress through the inhibition of radiation-induced NLRP3 inflammasome activation, thereby promoting the reconstruction of skin tissue 89. Porcine acellular dermal matrix hydrogels have been reported to effectively enhance the healing of RISI in rat models by modulating the inflammatory microenvironment, promoting angiogenesis, while simultaneously preventing excessive fibrosis and encouraging lipogenesis 90,91.

### 4.3 Nanomaterials

**Figure 5:**
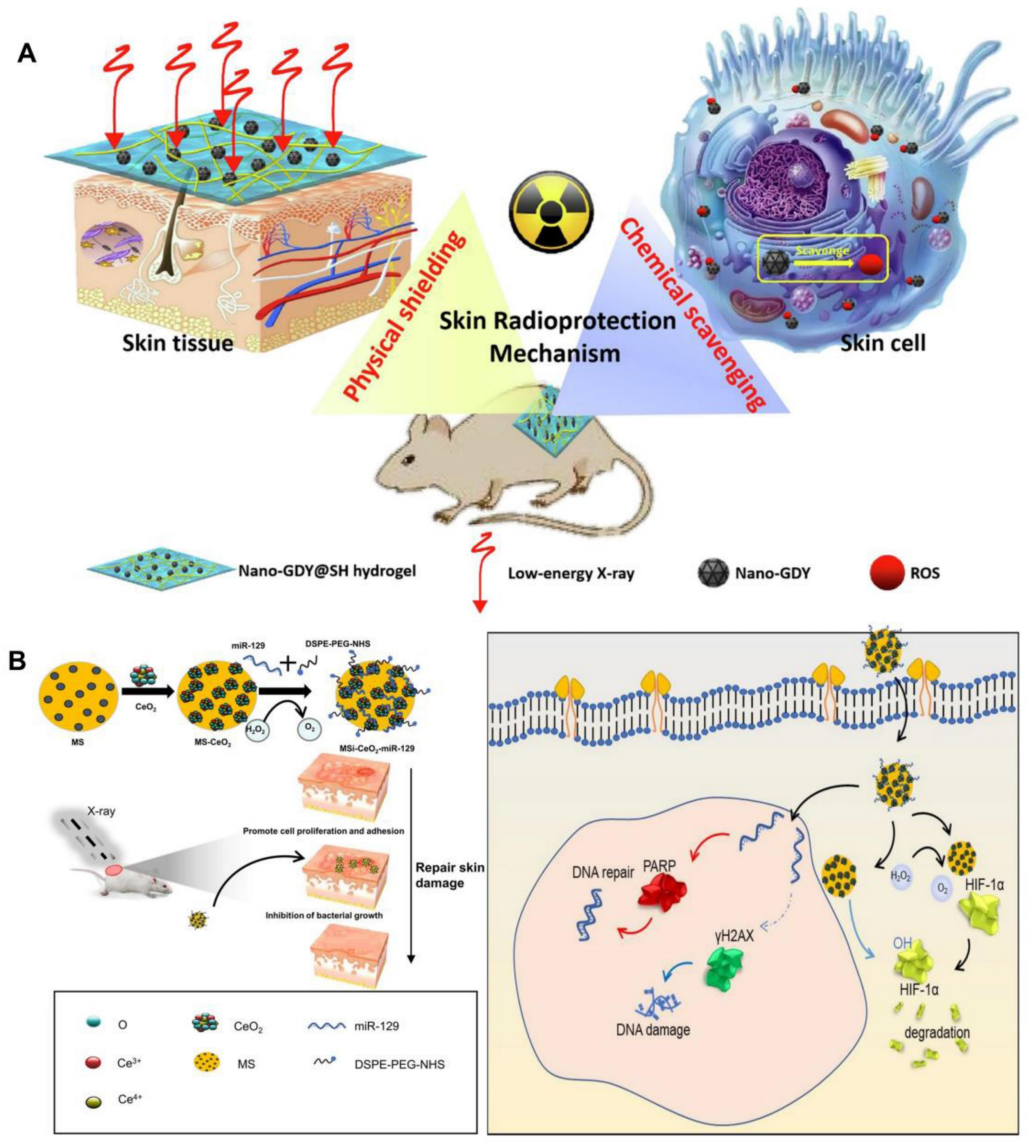
Nanomaterials in the therapeutic management of RISI: (A) The scheme of skin radioprotection by nano-GDY@SH hydrogel via both physically shielding of low-energy X-ray and chemically scavenging of broad-spectrum free radicals. Adapted with permission from 82, copyright 2022 Elsevier. (B) Schematic illustration showing the fabrication of MS-CeO_2_-miR129 and its application for RISI healing. Adapted with permission from 95, copyright 2022 Springer Nature.

With the rapid advancement of nanotechnology in the field of biology, the integration of multifunctional nanomaterials with hydrogels presents a promising new strategy for radiological protection dressings. On one hand, nanomaterials have demonstrated numerous advantages in radiation protection, including efficient and broad-spectrum free radical scavenging capabilities 122. On the other hand, due to the three-dimensional network structure inherent to hydrogels, they not only serve as carriers for nanoparticles but also possess significant potential for absorbing low-energy ionizing radiation owing to their high moisture retention properties 123. For instance, molybdenum disulfide (MoS_2_) nanosheets were extensively utilized in the biomedical field due to their large specific surface area and excellent biocompatibility, which enabled them to effectively eliminate various free radicals. Incorporating these nanosheets into hydrogels not only addressed the issue of poor mechanical properties of hydrogels but also imparted remarkable antioxidant capabilities, thereby enhancing the radiation protection effect on the skin 92. Similarly, due to the excellent chemical stability and broad-spectrum free radical scavenging ability of graphdiyne nanoparticles 93, sodium hyaluronate hydrogel loaded with it can effectively mitigate low-energy X-ray-induced skin edema and ulcers in mice, while also promoting wound healing **(Figure 5A)** 82. Graphene oxide had garnered attention as a wound repair material, particularly when combined with interferon-α-induced protein 6 (IFI6) hydrogel. This combination not only exhibited significant antibacterial properties but also reduced the radiation sensitivity of HaCaT cells, offering a promising solution for the repair of radioactive skin wounds 7. Fullerenols were recognized as "free radical sponges." The fullerenol-sodium hyaluronate hydrogel developed by Zhao *et al.* effectively mitigated ROS-induced damage and improved the viability of irradiated human keratinocytes, thereby alleviating radiation dermatitis 94. Zhou *et al.* utilized mesoporous silica to effectively anchor and disperse cerium oxide (CeO_2_) nanoparticles containing miR129 serum, resulting in the formation of nanocapsules with a distinctive core-shell structure. Notably, these nanocapsules demonstrate anti-reactive oxygen species (anti-ROS), anti-HIF-1α, and anti-radiation properties. This study underscores the clinical potential of miR129-loaded nanoparticles in modulating PARP and γH2AX, thereby reducing radiosensitivity and enhancing resistance to radiation-induced damage **(Figure 5B)** 95. Vascularization plays a critical role in tissue regeneration following RISI. Numerous studies have investigated the potential of nanoparticles loaded with vascular endothelial growth factor (VEGF) to promote angiogenesis during wound healing. To advance this strategy, Yu *et al.* developed a system utilizing chitosan (CS) nanoparticles encapsulated with VEGF-165. By modifying these nanoparticles with sodium tripolyphosphate (TPP), the system effectively packaged VEGF-165 and facilitated its sustained local release over an extended period. This approach significantly increased the concentration of VEGF-165, thereby enhancing microvascular remodeling and mitigating the progression of radioactive skin damage 96.

### 4.4 3D bioprinting

**Figure 6:**
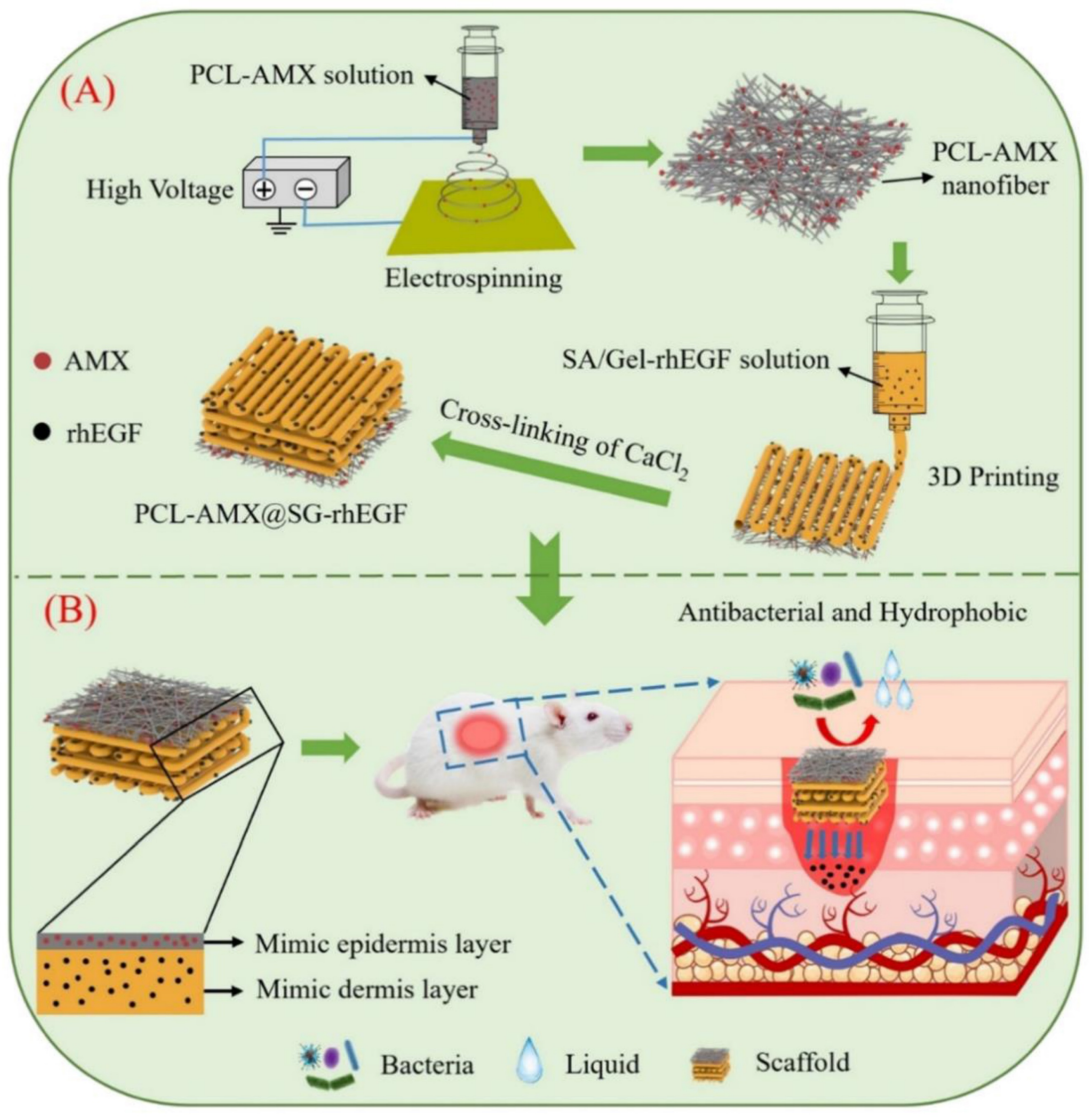
Schematic diagram depicting two aspects: (A) The preparation process of the scaffold; (B) The bioactive therapeutic mechanism of the scaffold in the wound area. Adapted with permission from 100, copyright 2024 Elsevier.

In recent years, bioprinting has demonstrated significant potential in the regeneration and repair of tissues and organs. In cases of irregular defects resulting from ionizing radiation, 3D printing technology can be tailored to meet the specific needs of individual patients. Furthermore, the customizable nature of printing inks combined with a variety of printing methods enhanced the effectiveness of treatments for RISI 124. Using bio-inks derived from the patient's own fibroblasts and keratinocytes, researchers developed personalized skin repair materials through 3D printing technology. These innovative materials were applied to patients suffering from radiation skin injuries. The results demonstrated that the bioprinted skin repair material significantly accelerated wound healing and effectively reduced inflammatory responses 97. The development of multi-layer composite materials designed to replicate the epidermis and dermis of the skin had yielded promising results. Song *et al.* incorporated amoxicillin into polycaprolactone nanofibers to create an antibacterial layer, while external human epidermal growth factor (rhEGF) was integrated with sodium alginate-gelatin for 3D printing as the inner layer of the scaffold. This innovative approach demonstrated remarkable drug release and antibacterial properties, significantly enhanced cell adhesion and proliferation, and effectively promoted wound healing **(Figure 6)** 100. Some researchers have suggested that isolating and culturing normal skin fibroblasts, followed by reprogramming them into induced pluripotent stem cells (iPSCs), represents a promising strategy for differentiating iPSCs into keratinocytes and endothelial cells. These differentiated cells can serve as seed cells for 3D bioprinted skin grafts. However, it is important to emphasize that the utilization of iPSCs necessitates the establishment of robust and standardized operating procedures 99. Chai *et al.* evaluated low-dose radiation-induced DNA damage and repair by 3D printing human foreskin fibroblast constructs 98. Although 3D bioprinting has found extensive application in the domains of tissue engineering and regenerative medicine, it continues to encounter numerous challenges. These challenges encompass aspects such as printing technology, bio-inks, and the selection and compatibility of cells. Furthermore, various factors influence the progression of skin radiation injuries, making early outcomes difficult to predict. It is essential to accurately assess the symptoms associated with skin damage in order to facilitate personalized treatment approaches. This may involve in-situ printing of cells and materials directly onto patients' wounds; however, this process is inherently complex. Additionally, ensuring sufficient vascularization of the engineered structures is crucial for maintaining the long-term viability of any bioprinted tissue construct. Without adequate vascularization, essential nutrients and gas exchange cannot be effectively achieved.

## 5. Summary and prospects

In this paper, we provide a comprehensive review of the mechanisms, clinical manifestations, treatment status, and research advancements concerning multifunctional biomaterials in the context of radiation-induced skin injury diseases. Despite significant progress made in recent years regarding the application of biomaterials for treating radiation skin injuries, several challenges persist that must be addressed before these innovations can be effectively translated into clinical practice:

(1) Complexity of the damage mechanism: The pathogenesis and injury mechanisms associated with various diseases represent critical guidelines for material design and warrant further investigation. The degree of radiation-induced reaction is not entirely dependent on the irradiation dose, but also closely related to the irradiation area and volume, which makes it difficult to judge the results in the early stage. Skin damage resulting from ionizing radiation occurs through two primary pathways: direct attack on DNA leading to its damage, and indirect harm caused by ROS generated when ionizing radiation interacts with water, which can also inflict damage on DNA and other cellular components. However, this description serves only as a preliminary overview. The pathogenesis of RISI is highly complex, resembling a chain reaction that encompasses multiple biological processes including injury signaling pathways, oxidative stress responses, inflammation, immune activation, and microvascular impairment. Consequently, researchers must transcend traditional notions of "target cells" to identify potential damaging factors. It is essential to delve deeper into molecular structures, cellular frameworks, and related signaling pathways in order to develop targeted therapeutic strategies 125. Therefore, a more comprehensive understanding of the mechanisms underlying radiation-induced tissue dysfunction is essential. This knowledge holds significant implications for the medical management of radiation injuries and informs the design and development of innovative, effective mechanism-based biomaterials.

(2) Safety of biomaterials: The safety of biomaterials* in vivo* is of paramount importance. Despite the vigorous advancement in biomaterial-mediated therapies and radiation protection research, substantiating their advantages for clinical applications remains in its early stages. Current efforts are primarily focused on the synthesis of multifunctional biomaterials, conducting *in vitro* experiments, and validating findings through animal models. Ongoing research must prioritize human specificity and safety assessments, as these are critical factors for enabling biomaterials to progress toward clinical applications. Consequently, when exploring novel biomaterials for the treatment of radiation skin injuries, implementing the following strategies may enhance their biosafety and efficacy: a) For the mechanisms of RISI, a comprehensive understanding of the interactions between biomaterials and cellular signaling pathways, as well as metabolic networks, is essential for designing biomaterials with targeted therapeutic activity 126. b) By employing interdisciplinary approaches and high-throughput screening techniques, novel categories of effective natural or synthetic materials are identified to enhance the therapeutic index 73. c) The specific nature of skin damage resulting from radiation must be thoroughly considered. For instance, the application of localized drug administration, along with percutaneous absorption and skin penetration, may enhance treatment efficacy. Additionally, it is important to take into account the antibacterial properties and adhesion characteristics of the materials used. d) The design of drug delivery systems for biomaterials must not only achieve high efficiency in drug loading but also ensure that the drug release rate is controllable. This is essential to prevent initial burst release or prolonged inefficient release. Furthermore, it is crucial for these systems to maintain stability during storage and transportation to avoid degradation or inactivation.

(3) Multifunctional design of biomaterials: While biomaterials have demonstrated significant advantages in the management of radioactive skin diseases, the clinical evidence supporting their superiority remains in its early stages. It is understood that the pathogenesis and clinical manifestations of RISI vary with increasing radiation dose and duration of irradiation, underscoring the importance of developing biological materials with targeted therapeutic applications. In the early acute phase of radiation damage, utilizing nanomaterials that possess anti-cellular senescence and antioxidant properties may represent a more effective approach 127. 3D-printed biomaterials enriched with highly active ingredients not only facilitate personalized customization but also aid in regulating inflammatory responses and promoting tissue repair during the intermediate stages of injury 128. The moist environment provided by hydrogels enhances subsequent wound healing and can help mitigate scar formation 129. However, it is important to recognize that the functionality of individual biomaterials is inherently limited; thus, optimizing composite designs for these materials is essential. Factors such as DNA damage repair, cellular aging delay, varying effects across different stages of radiation-induced skin injury, simultaneous drug loading and controlled release functionalities, as well as microenvironment regulation within irradiated skin wound tissues must all be considered in the design process for biomaterials 130. Consequently, extensive research and clinical trials are essential to investigate various biomaterials and ascertain their critical role in RISI therapy.

(4) Efficacy of clinical treatment: Although biomaterials have been extensively evaluated for safety in animal models, clinical studies remain preliminary. Currently, several urgent questions persist regarding the toxicity and systemic immune responses induced by these biomaterials, their mechanisms of action in radiation-induced diseases, and the efficacy of protective measures-issues that require further investigation by researchers. Given the unique characteristics of various organs and tissues, the severity of radiation-induced diseases exhibits significant heterogeneity. Furthermore, most studies on biomaterials have predominantly focused on tissues and organs such as the skin, gastrointestinal tract, and lungs 131-133. It is crucial to broaden research efforts to encompass other clinically relevant sites affected by radiation damage, including the eyes, liver, bladder, and hematopoietic system. It is crucial to emphasize the necessity of conducting a comprehensive evaluation of biological materials, and this process must be standardized. First, by leveraging bionic principles, we can design materials with enhanced biocompatibility, thereby improving therapeutic outcomes. Second, the integration of artificial intelligence with bioprinting techniques holds promise for producing biomaterial structures with greater specificity, thus paving the way for personalized therapeutic interventions. Finally, employing organoid models may serve as an effective preclinical tool for assessing the performance of biomaterials, thereby facilitating advancements in the management of radiation-induced diseases.

In summary, the rapid advancements in materials science and regenerative medicine have led to the discovery of new biomaterials that exhibit remarkable capabilities in antioxidant activity, cell proliferation, immune regulation, and angiogenesis. These biomaterials hold significant potential for the treatment and protection of diseases associated with radiation damage. Consequently, the future clinical application and commercialization of smart biomaterials remain highly anticipated. However, achieving success necessitates further verification and evaluation of these systems. It is imperative to strive towards providing more effective and safer solutions for managing radiation-induced diseases and enhancing radiation protection at the earliest opportunity.

## Figures and Tables

**Table 1:** Classification and manifestation of acute radiation-induced skin injury

Damage grade	Changes in the skin
Grade 0	Unchanged
Grade I	Erythema or dry desquamation
Grade Ⅱ	Moderate erythema or flaky wet desquamation
Grade Ⅲ	Fused wet desquamation or pitted edema with a diameter ≥1.5 cm, not limited to the fold
Grade Ⅳ	Full dermal skin necrosis or ulceration

**Table 2:** Classification and manifestation of chronic radiation-induced skin injury

Damage grade	Changes in the skin
Grade 0	Unchanged
Grade I	Mild atrophy, hyperpigmentation, slight hair loss
Grade Ⅱ	Lamellar atrophy, moderate capillaries dilation, complete hair exfoliation
Grade Ⅲ	Marked atrophy, marked telangiectasia
Grade Ⅳ	Ulceration
Grade Ⅴ	Died as a direct result of a late radioactive reaction

**Table 3:** Studies on the drug therapy and cytokines to the treatment of radiation-induced skin injury

Treatment methods	Composition	Functions	Limitations	Ref
Drug therapy	Superoxide dismutase	Scavenging free radicals	Limited routes of administration, poor stability	53
	Corticosteroids	Anti-inflammatory, antioxidant, immunosuppressive	Long-term use is easy to cause skin atrophy	54
	Statins	Antibacterial, anti-inflammatory, antioxidant	Lack of substantial clinical evidence	55
	Melatonin	Scavenging free radicals, inhibiting apoptosis	Lack of large-scale, randomized controlled trials	56
	Amifostine	Antioxidant	Obvious side effects (nausea, vomiting, fatigue, etc.)	57
	Triethanolamine	Recruit macrophages, stimulate granulation tissue formation	Potential irritation, allergic reactions	54
	Sucralfate	Antibacterial, anti-inflammatory, promote angiogenesis	The clinical efficacy is controversial	58
	Cordycepin	Anti-DNA damage, anti-cell aging	Low bioavailability, high cost	59
	Quercetin	Anti-DNA damage, removal of senescent cells, antioxidation	Difficult to extract and purify	61
Cytokines	EGF	Promote re-epithelialization	High cost, easy inactivation	63
	FGF	Enhance angiogenesis		65
	rhGM-CSF	Anti-infection, promote wound healing		66

**Table 4:** Biomaterial-based treatment strategies for radiation-induced skin injury

Strategy	Composition	Functions	Limitations	Ref
Stem Cells and exosomes	ADSCs	Anti-inflammatory, inhibit skin fibrosis, prevent irregular collagen deposition	Immunogenicity, tumorigenic risk	74
	BMSCs	Reduce inflammation and fibrosis		75
	iPSCs	Promote re-epithelialization, enhance angiogenesis		76
	Natural Exosomes	Promote the proliferation and migration of epithelial cells, inhibit the apoptosis of vascular endothelial cells	Undefined composition, difficult to extract and purify	74
	Engineering Exosomes	Targeted therapy		77
Hydrogels	Gallic acid	Antioxidant	Poor stability, limited skin permeability	78
	EGCG	Antioxidant, promote angiogenesis		79
	Tannic acid	Tissue adhesion, free radical scavenging, immune regulation		80
	Curcumin	Antioxidant, anti-inflammatory	Poor solubility	80
	Deferoxamine	Reduce inflammation, promote angiogenesis	Potential cytotoxicity	36
	Retinoic acid	Anti-inflammatory, accelerate fibrocyte proliferation	Long-term use can damage the skin barrier	36
	Hyaluronic acid	Reduce inflammation, maintain cell activity, relieve pain	Poor mechanical property	81
	Gelatin Methacryloyl	Physical absorption of low energy X-rays		82
	Sodium alginate	Antibacterial, promote angiogenesis		83
	Chitosan	Antibacterial, enhance cell adhesion		84
	Keratin	Promote keratinocyte and fibroblast migration, reduce inflammation	Extraction complexity	85
	Polypeptide	Antioxidant, promote epidermal tissue regeneration and angiogenesis	High cost	86,87
	Glucomannan	Regulate the polarization of macrophages towards M2 phenotype	Poor water solubility	88
	Sildenafil citrate	Promote re-epithelialization, vascularization and granulation tissue formation	Insufficient mechanical strength	88
	Ferulic acid	Antioxidant, inhibition of NLRP3 inflammasome activation to reduce inflammation	The optimal therapeutic concentration is unclear	89
	Acellular Dermal Matrix	Regulate macrophage polarization, enhance angiogenesis, prevent excessive fibrosis, promote lipogenesis	High cost, limited mechanical properties, potential immunogenicity	90,91
Nanomaterials	Molybdenum disulfide	Scavenging free radicals	Poor dispersity, long-term safety to be verified	92
	Graphdiyne	Good chemical stability, broad-spectrum free radical scavenging ability		93
	Graphene oxide	Antibacterial, reduce the radiation sensitivity of HaCaT cells		7
	Fullerenols	Antioxidant		94
	Silicon dioxide	Antioxidant		95
	Cerium oxide	Antioxidant		95
	VEGF-chitosan nanoparticles	Protect vascular endothelial cells, promote angiogenesis	High cost, relatively complex process	96
3D printing	Fibroblasts	Reduce inflammation, assess DNA damage	Time-consuming, complex to operate	97,98
	Keratinocytes	Anti-inflammatory, promote wound healing		97
	Endothelial cells	Vascular remodeling		99
	Polycaprolactone encapsulated amoxicillin	Antibacterial	Uncontrollable release rate and timing	100
	Sodium alginate-gelatin encapsulated rhEGF	Enhance cell proliferation and adhesion		100

**Table 5:** The response elements, mechanisms and treatment effects of intelligent response hydrogels

Response element	Example	Assembly mechanism	Treatment effect	Ref
Temperature responsive	Pluronic F127	Composed of hydrophilic poly(ethylene oxide) (PEO) blocks at both ends and a hydrophobic poly(propylene oxide) (PPO) block in the middle	Stimulate the expression of vascular endothelial growth factor (VEGF) and transforming growth factor-β1 (TGF-β1) to promote wound healing	108
Photothermal-responsive	Polydopamine	A lot of phenolic hydroxyl groups	Antioxidant, anti-inflammatory	117
pH-responsive	Alginate grafted with dopamine	Amidation	Antibacterial, regulate ROS, promote cell proliferation	118
H_2_O_2_-responsive	L-arginine- coupled chitosan	Amidation	Promote angiogenesis and collagen deposition	119
Enzyme responsive	Multi-arm PEG	The function of matrix metalloproteinases	Reduce inflammation	111,120
Glucose-responsive	phenylboronic acid	Boronic ester bonds	Anti-inflammatory, promote the secretion of VEGF	121
